# Increased levels of circulating cell‐free double‐stranded nucleic acids in the plasma of glioblastoma patients

**DOI:** 10.1002/jex2.168

**Published:** 2024-08-02

**Authors:** Elisabeth Rackles, Elena Zaccheroni, Patricia Hernandez Lopez, Stefania Faletti, Massimiliano Del Bene, Francesco DiMeco, Giuliana Pelicci, Juan M Falcon‐Perez

**Affiliations:** ^1^ Center for Cooperative Research in Biosciences (CIC bioGUNE) Basque Research and Technology Alliance (BRTA) Derio Spain; ^2^ Department of Experimental Oncology European Institute of Oncology (IEO) IRCCS Milan Italy; ^3^ Department of Neurosurgery Fondazione IRCCS Istituto Neurologico Carlo Besta Milan Italy; ^4^ Department of Pathophysiology and Transplantation University of Milan Milan Italy; ^5^ Department of Neurological Surgery Johns Hopkins Medical School Baltimore Maryland USA; ^6^ Department of Translational Medicine University of Piemonte Orientale Novara Italy; ^7^ Centro de Investigación Biomédica en Red de Enfermedades Hepáticas y Digestivas (Ciberehd) Madrid Spain; ^8^ Ikerbasque, Basque Foundation for Science Bilbao Spain

**Keywords:** cell‐free nucleic acids, circulating tumour DNA, extracellular RNA, extracellular vesicles, flow cytometry, glioblastoma, Pyronin Y

## Abstract

Circulating cell‐free nucleic acids are considered a promising source of biomarkers for diseases and cancer. Liquid biopsy biomarkers for brain tumours represent a major, still unmet, clinical need. In plasma, nucleic acids can be free or be associated with extracellular vesicles (EVs). Here we report an easy and reproducible method to analyse cell‐free nucleic acids in plasma and EVs by conventional flow cytometry easy to translate into the clinics. Nucleic acids associated with the EVs or present in plasma samples are stained by Pyronin Y, which is a fluorescent dye that is preferably binding double‐stranded nucleic acids. Fluorescent staining of EVs isolated from cell‐conditioned media is suitable for DNA and RNA detection by flow cytometry. The nucleic acids are partially protected from degradation by the EVs’ membrane. Additionally, DNA and RNA can be stained in plasma samples and plasma‐derived EVs. Remarkably, analysis of plasma from patients and healthy individuals reveals a difference in their nucleic acid profiles. Taken together, our results indicate that the proposed methodology, which is based on conventional direct flow cytometry, is a promising easy tool for plasma nucleic acid analysis.

## Graphical abstract

We describe a new staining method to detect nucleic acids in extracellular vesicles and human plasma samples by conventional flow cytometry. We show that plasma samples from glioblastoma patients and healthy individuals display different nucleic acid profiles. Our quick two‐steps method detects nucleic acids in extracellular vesicles and in small volumes of plasma samples which offers a sensitive and low invasive method for routine evaluation of patients.

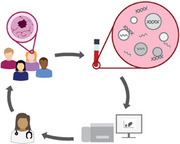

## 1 INTRODUCTION

The analysis of circulating cell‐free nucleic acids is an emerging field for the identification of biomarkers for cancer. Many studies focus on biomarker detection in blood samples since it is a minimally invasive source for liquid biopsy. For the identification of new biomarkers, the cell‐free nucleic acids are analysed using mainly sequencing‐ or PCR‐based methods. Albeit the analysis of cell‐free nucleic acids is promising for cancer diagnosis, it faces limitations like insufficient sensitivity (García‐Pardo et al., 2022). Thus, improved methods for the analysis of cell‐free nucleic acids would enhance their potential application as biomarkers.

Examples of cell‐free nucleic acids are double‐stranded nuclear DNA, mitochondrial DNA, messenger RNA and noncoding RNA (Cheung et al., 2019; Kustanovich et al., 2019). The nucleic acids can be associated with extracellular vesicles (EVs) (Ghanam et al., 2022; Wu et al., 2022) or extracellular particles (Zhang et al., 2018). EVs are surrounded by a membrane while extracellular particles are not membrane surrounded (Zhang et al., 2018). EV‐associated DNA is comprised of single‐stranded DNA, double‐stranded DNA and mitochondrial DNA, with double‐stranded DNA being the most abundant form (Balaj et al., 2011; Guescini et al., 2010; Liu et al., 2022; Thakur et al., 2014). Most of the DNA is present on the surface of the EVs (Liu et al., 2022; Neuberger et al., 2021). Furthermore, EVs contain all types of RNA. EVs participate in intercellular communication by the transfer of their cargo from the secretory cell to a recipient cell. The EV's cargo is not only comprised of nucleic acids but also proteins and metabolites and its molecular composition varies depending on the biological and pathological state of the secretory cell (Rackles et al., 2022). These characteristics suggest that the EV's cargo can serve as a source of reliable biomarkers for different diseases.

In this frame, brain tumours represent the ideal setting for biomarker development. Diagnosis and treatment are guided by imaging and surgery which are limited by sub‐optimal accuracy and introduce risks for patients. As a matter of fact, liquid biopsy represents a major, still unmet, clinical need (Gatto et al., 2021). Among brain tumours, glioblastoma (GBM) is the most common and lethal sub‐type, characterized by impressive intra‐ and inter‐tumour heterogeneity and invasiveness which make any treatment virtually ineffective (Becker et al., 2021). The analysis of micro RNAs as biomarkers in liquid biopsy of GBM patients has achieved promising results. For instance, the analysis of the levels of miR‐210 in EVs or serum is a potential biomarker for the diagnosis and prognosis stratification of glioma (Lai et al., 2015; Lan et al., 2020). In contrast, the analysis of cell‐free DNA is more challenging. Indeed, the percentage of detection of genetic alterations in plasma samples varies between 27 and 55% (Piccioni et al., 2019; Schwaederle et al., 2016).

In the effort to overcome these limitations, we developed a new method for the analysis of cell‐free nucleic acids in plasma samples from GBM patients. In particular, while most biomarker studies focus on the analysis of specific nucleic acids, our approach addresses an easy tool to detect the total nucleic acid content of plasma. As stated above, GBM presents very low levels of detectable cell‐free DNA in plasma (Bettegowda et al., 2014). Technically, free nucleic acids have a limited half‐life, which varies between several minutes to few hours (Kustanovich et al., 2019). In contrast, molecules present in EVs are protected by the EV's membrane and can be enriched by the isolation of the EVs from plasma (Ghanam et al., 2022). Thus, we aimed to study the EVs‐associated nucleic acids with a novel method based on staining with Pyronin Y which is a membrane‐permeable dye that intercalates in double‐stranded DNA and double‐stranded RNA (Darzynkiewicz et al., 1987). After staining, EVs are analysed using flow cytometry. The novelty of our staining methodology resides in being applicable not only to EVs‐associated nucleic acids, but also to total cell‐free nucleic acids in plasma at the same time. We applied the Pyronin Y staining to plasma samples of GBM patients and healthy individuals to observe differences in their total cell‐free nucleic acids profiles.

## 2 RESULTS

### 2.1 Pyronin Y staining is suitable for the detection of EVs by conventional flow cytometry

**FIGURE 1:**
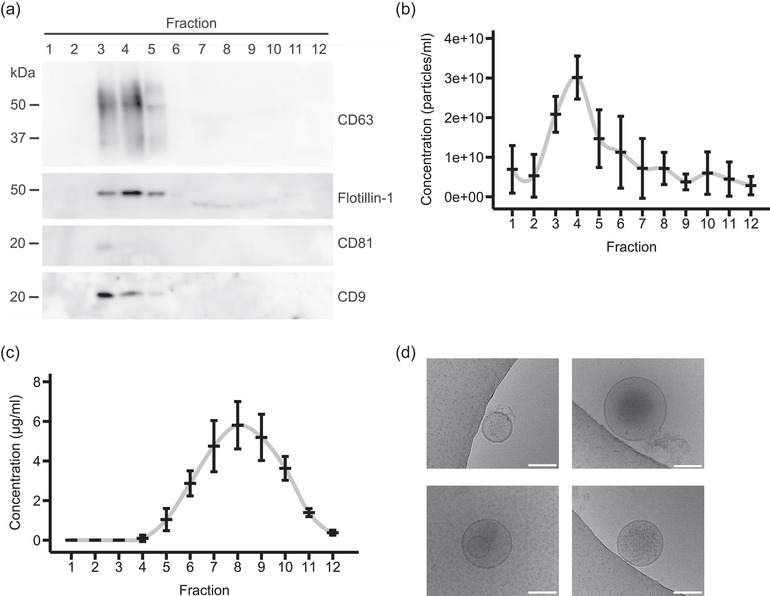
Characterization of EVs isolated from U‐87 MG cell‐conditioned media by SEC. (a) Immunoblots of the analysis of all SEC fractions. Representative images of three independent isolations are shown (*n* = 3). (b) Particle concentration of each fraction measured by NTA. Mean and standard deviation of three independent experiments are shown (*n* = 3). (c) Protein concentration of each fraction measured by Bradford assay. Mean and standard deviation of three independent experiments are shown (*n* = 3). (d) Representative images of cryo‐electron microscopy of EVs present in fraction four. The scale bar represents 100 nm.

To establish the analysis of nucleic acids associated with EVs, we isolated EVs from U‐87 MG cell‐conditioned media by size exclusion chromatography (SEC). EVs were mostly present in fractions three to five as confirmed by the presence of the EV markers CD9, CD63, CD81, and Flotillin‐1 (Figure 1a) and by the absence of the cellular marker COX IV (Figure S1a). Furthermore, nanoparticle tracking analysis showed the highest number of particles in fraction four, while proteins eluted in the later fractions of the column as shown by a Bradford assay (Figures 1b and 1c). Cryo‐electron microscopy showed EVs of various morphology and sizes between about 50 and 250 nm (Figures 1d and S1b). Some vesicles showed double lipid bilayers and electron‐dense cargoes.

**FIGURE 2:**
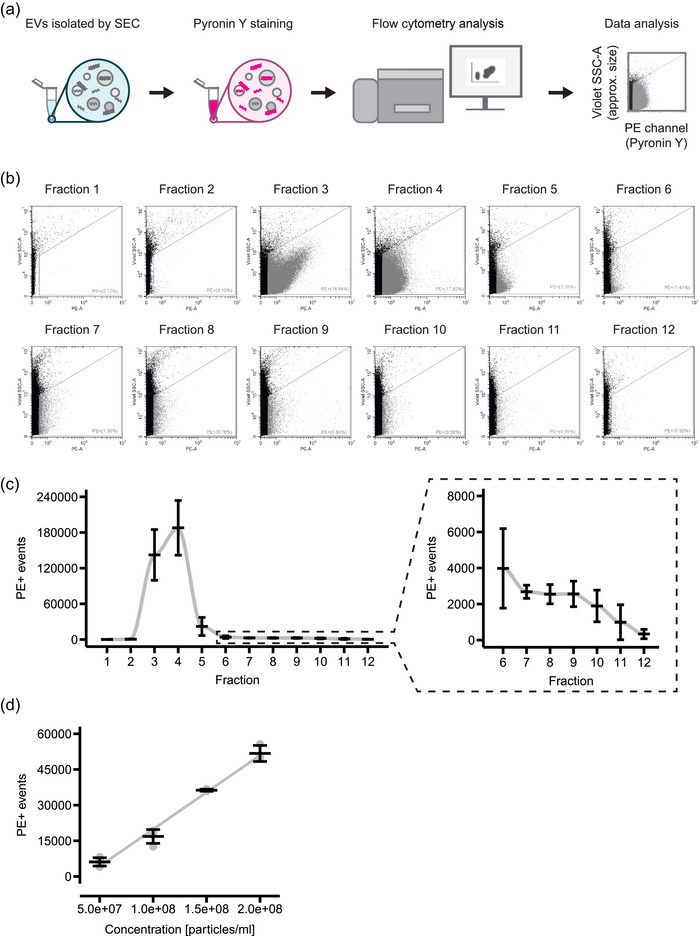
Pyronin Y staining of EVs isolated from U‐87 MG cell‐conditioned media by SEC. (a) Schematic of the workflow. (b) Representative dot plots of all SEC fractions stained with Pyronin Y and analysed by flow cytometry. (c) Quantification of the number of PE+ events of each fraction. Mean and standard deviation of three independent experiments are shown (*n* = 3). (d) Staining of U‐87 MG EVs (pooled fractions 3–5) and quantification of the number of PE+ events dependent on the particle concentration. Mean and standard deviation of three technical replicates is shown (*n* = 3). EVs, extracellular vesicles; SEC, size exclusion chromatography.

Pyronin Y is a membrane‐permeable dye that intercalates in double‐stranded DNA and double‐stranded RNA and fluoresces at an orange‐red wavelength (Darzynkiewicz et al., 1987). Due to these properties, we used Pyronin Y to analyse the total nucleic acid content of EVs by flow cytometry. The experimental workflow was comprised of only three steps: (i) isolation of EVs by SEC, (ii) incubation of a fraction of the sample in Pyronin Y staining solution, (iii) direct analysis using a conventional flow cytometry (Figure 2a). Before we analysed EVs, we stained media incubated without cells with Pyronin Y to exclude potential background signal from the cell media. Flow cytometry analysis only showed some PE signal in events with high violet SSC‐A (S2a ). The violet SSC‐A is an approximation for the size of the particles as shown by the analysis of fluorescent beads with standardized diameter (Figure S2b). Therefore, we used a gating strategy for PE+ events that excluded these large background events (approx. >200 nm). Next, we stained all fractions of the SEC of the U‐87 MG cell‐conditioned media with Pyronin Y to analyse which fractions have the highest nucleic acid content. We found high numbers of PE+ events in fractions three and four (Figures 2b and 2c), which coincides with the fractions containing EVs (Figure 1). For the following experiments, we pooled the EV containing fractions three to five, hereafter referred to as EVs. Unstained EVs did not show any PE+ signal and treatment of the EVs with Triton X‐100 reduced the number of events (Figures S2c and S2d). Comparison of the PE+ events of the EVs with fluorescent beads with standardized diameter revealed that the PE+ events of the EVs displayed a range of violet SSC‐A that mostly corresponded to beads smaller than 200 nm (Figure S2b). To ensure that we detect the EVs as single events and to exclude so‐called ‘swarm effects’ (Welsh et al., 2020; Welsh et al., 2023), we stained a serial dilution of EVs. With increasing EV concentrations from 5*10^7^ to 2*10^8^ particles/Ml, the number of PE+ events also increased (*R* ^2 ^= 0.97) (Figure 2d). To test if Pyronin Y is indeed staining nucleic acids associated with EVs, we performed a double staining with the EV marker CD63. The EVs showed a population that was positive for the CD63 antibody (Figure S2e). Interestingly, especially the events with a high PE+ intensity were positive for CD63. Taken together, we can be confident that Pyronin Y can be used for the detection of EVs using a conventional flow cytometer.

### 2.2 Pyronin Y stains DNA and RNA associated with EVs

**FIGURE 3:**
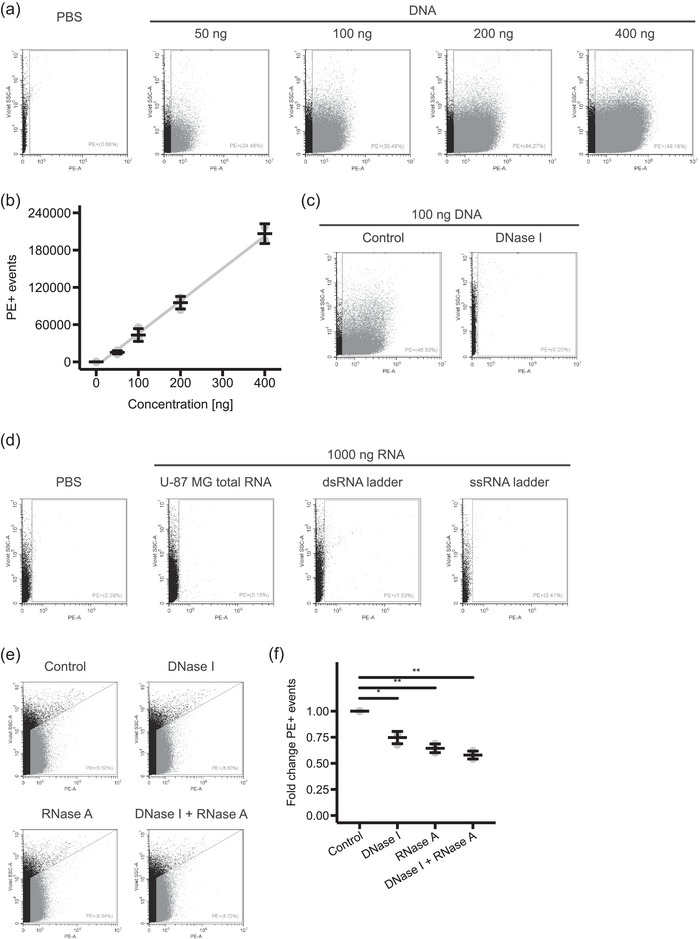
DNA and RNA specificity of Pyronin Y staining. (a) Representative dot plots of PBS or different amounts of DNA stained with Pyronin Y and analysed by flow cytometry. (b) Quantification of the number of PE+ events dependent on the DNA concentration. Mean and standard deviation of three independent experiments are shown (*n* = 3). (c) Representative dot plots of DNA with or without DNase I treatment, stained with Pyronin Y and analysed by flow cytometry. (d) Representative dot plots of PBS or RNA stained with Pyronin Y and analysed by flow cytometry. (e), (f) U‐87 MG cell‐conditioned media derived EVs were subjected to DNase I, RNase A or combined DNase I and RNase A treatments, stained with Pyronin Y and analysed by flow cytometry. (e) Representative dot plots. (f) Quantification of the number of PE+ events. Mean and standard deviation of three independent experiments are shown (*n* = 3). *p < 0.05 and **p < 0.01 by a one sample *t*‐test.

Next, we asked if the Pyronin Y staining of EVs is specific to nucleic acids. First, we tested if Pyronin Y can stain free DNA and RNA. We found that genomic DNA from U‐87 MG cells can be stained with high specificity. Flow cytometry analysis of stained DNA showed a strong fluorescent signal in the PE channel and the number of PE+ events showed a linear correlation with the DNA concentration (*R* ^2 ^= 0.95) (Figures 3a and 3b). DNase I digest completely removed the signal confirming the DNA specificity of the staining (Figure 3c). In contrast, we did not observe a staining of total RNA from U‐87 MG cells, double‐stranded RNA ladder or single‐stranded RNA ladder (Figure 3d).

Next, we asked if the observed PE+ signal of the stained EVs (Figure 2) was only due to DNA or also due to RNA associated with the EVs. To this end, we treated the EVs with DNase I or RNase A before they were stained with Pyronin Y. Nucleic acids on the surface of the EVs would be degraded leading to a decrease in PE+ events. In contrast, if Pyronin Y stains nucleic acids that are inside the EV's lumen, they would be protected from degradation by DNase I or RNase A. Treatment of the EVs with DNase I led to a partial decrease in PE+ events showing that 25% of the stained DNA is present outside of the EV (Figures 3e and 3f). Similarly, RNase A treatment led to a decrease in PE+ events of 36%. Double treatment with DNase I and RNase A decreased the PE+ events by 42%. To summarize, the nucleic acids are partially associated with EV's surface and partially present inside the EV. Since we found that only Pyronin Y staining of RNA associated with EVs was possible, we hypothesize that we were not able to visualize free RNA as it might be below the detection limit of our flow cytometer.

### 2.3 Pyronin Y stains nucleic acids in plasma

**FIGURE 4:**
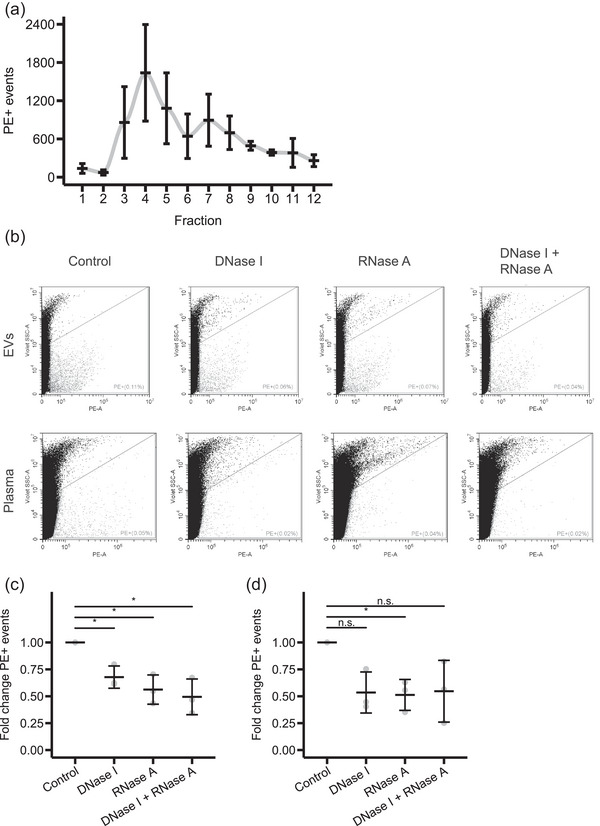
Pyronin Y staining of plasma samples and plasma EVs of healthy individuals. (a) Isolation of EVs from plasma by SEC and subsequent Pyronin Y staining of each fraction. Quantification of the number of PE+ events of each fraction by flow cytometry. Mean and standard deviation of SEC of plasma samples from three donors are shown (*n* = 3). (b)–(d) Plasma EVs and plasma samples were subjected to DNase I, RNase A or combined DNase I and RNase A treatments, stained with Pyronin Y and analysed by flow cytometry. (b) Representative dot plots. (c) Quantification of the number of PE+ events of plasma EVs. Mean and standard deviation of three independent experiments are shown (*n* = 3). *p < 0.05 by a one‐sample *t*‐test. (d) Quantification of the number of PE+ events of plasma samples. Mean and standard deviation of three independent experiments are shown (*n* = 3). *p < 0.05 and n.s. = not significant by a one‐sample *t*‐test.

Our goal is to analyse the total nucleic acid content of plasma‐derived EVs hypothesizing that conventional flow cytometry could be a functional and simple technique for that. Therefore, we isolated EVs from human reference plasma by SEC to analyse them by Pyronin Y staining. Like for cell‐conditioned media, the fractions with high particle concentration and low protein concentration were fractions three to five (Figures S3a and S3b). These fractions also showed the highest number of PE+ events (Figure 4a). We stained the pooled EV fractions three to five with Pyronin Y and observed PE+ events that were partially sensitive to DNase I or RNase A treatment and were reduced by 32 and 44%, respectively (Figures 4b and 4c).

We observed that the later SEC fractions six to 12 also showed PE+ events (Figure 4a) and, therefore, hypothesized that the analysis of isolated EVs might lead to a loss of valuable information by excluding the cell‐free nucleic acids that are present in the later fractions from the analysis. Thus, we tested if we could also directly stain plasma samples. This would not only reduce the loss of information due to the analysis of selected fractions but would also reduce sample processing. We stained plasma with Pyronin Y and found that the PE+ events were also sensitive to DNase I or RNase A treatment (Figures 4b and 4d). However, the treatments showed more variability than the treated EVs. Unstained plasma did not show any PE+ events, confirming that the fluorescence is from the Pyronin Y staining (Figure S3c). To summarize, Pyronin Y staining can also be applied to diluted plasma samples without prior isolation of EVs.

### 2.4 Pyronin Y staining of plasma samples is highly reproducible

**FIGURE 5:**
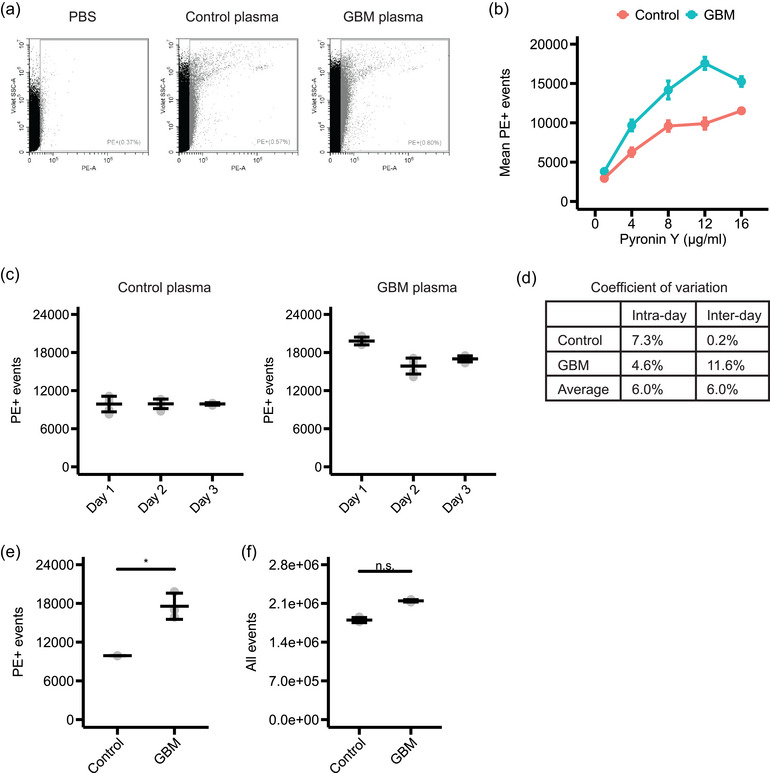
Optimization of the Pyronin Y staining of plasma samples of GBM patients and healthy controls. (a)–(f) Pooled plasma samples were stained with Pyronin Y and analysed by flow cytometry in five technical replicates in three independent experiments. (a) Representative dot plots of plasma samples stained with a Pyronin Y concentration of 12 μg/mL. (b) Quantification of the number of PE+ events dependent on the Pyronin Y concentration. Mean and standard deviation of three independent experiments are shown (*n* = 3). (c)–(f) Analysis of plasma samples stained with a Pyronin Y concentration of 12 μg/mL. (c) Quantification of the number of PE+ events. Mean and standard deviation of five technical replicates are shown (*n* = 5). (d) Coefficient of variation of the analysis of the five technical replicates (intra‐day) and three independent experiments (inter‐day). (e) Quantification of the number of PE+ events. Mean and standard deviation of three independent experiments are shown (*n* = 3). *p < 0.05 by a two‐sample *t*‐test. (f) Quantification of the total number of events. Mean and standard deviation of three independent experiments are shown (*n* = 3). n.s. = not significant by a two‐sample *t*‐test.

We aimed to identify differences in the nucleic acid content of plasma from GBM patients and healthy controls. First, we tested which Pyronin Y concentration was most suitable for the staining of the plasma samples. We tested concentrations between 1 and 16 μg/mL. We found that using a 12‐μg/mL staining solution shows the largest difference in PE+ events between GBM patients and healthy controls (Figures 5a and 5b). To assess the reproducibility of the staining method, we tested the intra‐ and inter‐day variability of technical replicates. We stained pooled plasma samples of nine GBM patients or nine healthy controls with 12‐μg/mL Pyronin Y and analysed the samples in five technical replicates on 3 different days. The mean coefficient of variation of the technical replicates was 7.3% for control samples and 4.6% for GBM samples (Figures 5c and 5d). The mean coefficient of variation between days was 0.2 and 11.6%, respectively. Additionally, we found that the pooled plasma samples of GBM patients showed significantly more PE+ events than the pooled plasma samples of healthy controls (Figure 5e). Furthermore, the total number of events was slightly but not significantly increased in GBM samples (Figure 5f). Taken together, we optimized the Pyronin Y staining of plasma samples which reveals with high reproducibility an increased nucleic acids content for GBM patients.

### 2.5 Pyronin Y staining reveals differences in the nucleic acid content of plasma from GBM patients

**FIGURE 6:**
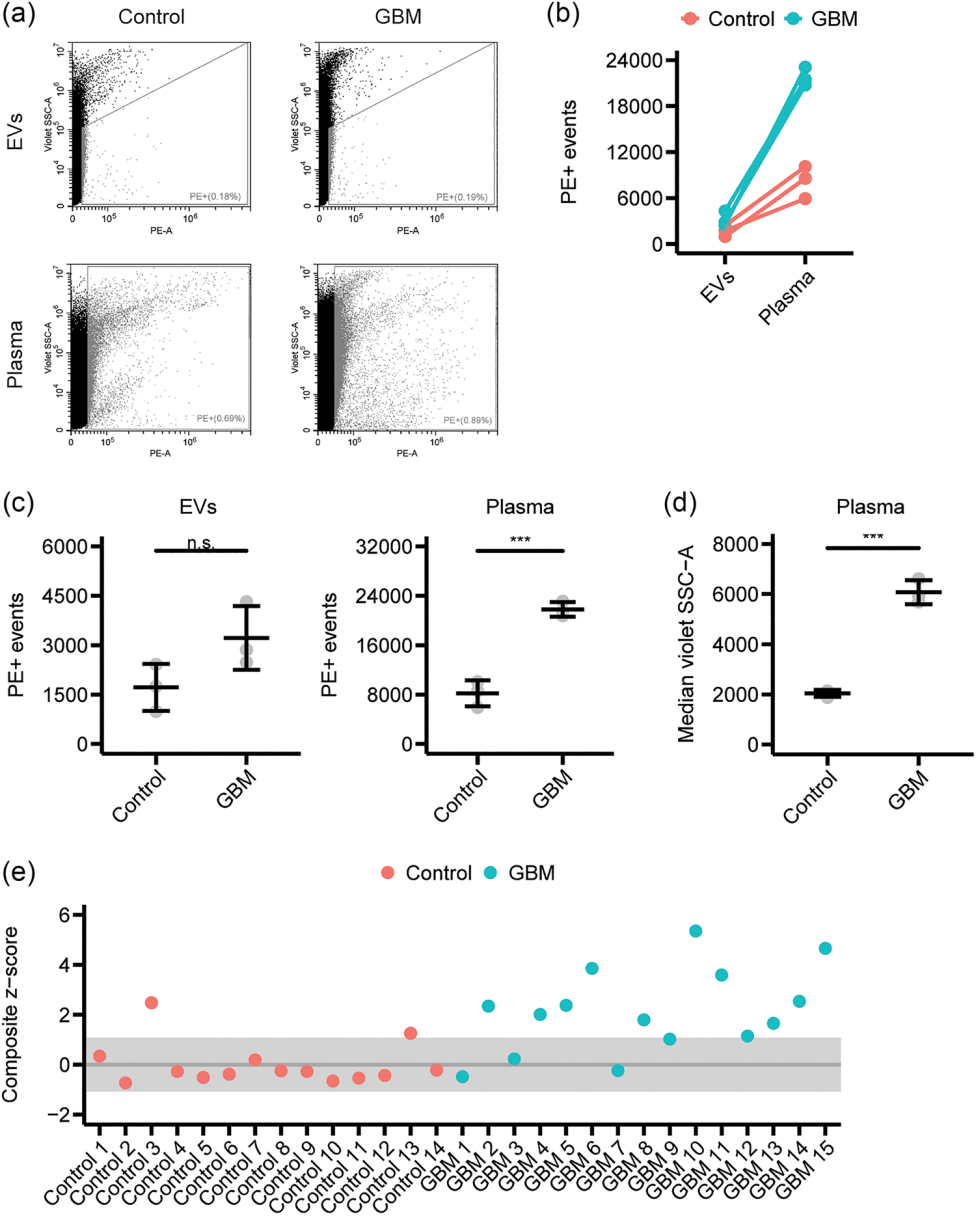
Pyronin Y staining of plasma samples of GBM patients and healthy controls. (a)–(c) EVs were isolated from three plasma samples per group. The EVs and the respective plasma samples were stained with Pyronin Y and analysed by flow cytometry. (a) Representative dot plots of stained EVs and plasma samples. (b) Quantification of the number of PE+ events of EVs and plasma samples. (c) Quantification of the number of PE+ events of EVs and plasma samples. Mean and standard deviation of three independent samples are shown (*n* = 3). ***p < 0.001 and n.s. = not significant by a two‐sample *t*‐test. (d) Quantification of the median violet SSC‐A of plasma samples. Mean and standard deviation of three independent samples are shown (*n* = 3). ***p < 0.001 by a two‐sample *t*‐test. (e) Composite *z*‐score of the number of PE+ events and violet SCC‐A of plasma samples stained with Pyronin Y and analysed by flow cytometry.

We asked if the difference in nucleic acid content between plasma samples of GBM patients and healthy controls is also reflected in EVs. We isolated EVs from plasma samples of three GBM patients and three healthy controls and stained the EVs and the respective plasma samples with Pyronin Y (Figure 6a). EVs and plasma samples showed the same trend that GBM patients have increased levels of nucleic acids (Figure 6b). However, statistical analysis showed that the nucleic acids content in GBM patients is only significantly increased in plasma samples and not in EVs (Figure 6c). This indicates that for the discrimination of GBM and control samples, it is better to analyse plasma samples than isolating EVs from the plasma thereby losing valuable information of free nucleic acids that are not associated with EVs. Analysing all events, we found that the median violet SSC‐A (which is an approximation for the size (Figure S2b)) is significantly increased in plasma samples of GBM patients (Figure 6d). This suggests that not only the number of PE+ events but also the median violet SSC‐A of the events is useful to discriminate between GBM patients and healthy controls.

Finally, we applied our new method to a cohort of 15 GBM patients and 14 healthy controls. We stained the plasma samples with Pyronin Y and measured them in five technical replicates. We calculated a composite *z*‐score of the violet SSC‐A of all events and the number of PE+ events relative to the control samples. Most of the control samples (86%) had a *z*‐score between −1 and 1 (Figure 6e). In contrast, 80% of the GBM samples had a *z*‐score > 1. Thus, most GBM patients show an increased number of PE+ events and a larger violet SSC‐A compared to healthy controls. The calculation of separate *z*‐scores for violet SSC‐A and PE+ events showed that GBM and control samples had a higher difference in the violet SSC‐A than in the number of PE+ events (Figure S4). To summarize, this experiment suggests that Pyronin Y staining of plasma samples can be used to detect differences between GBM patients and healthy individuals using conventional flow cytometry.

## 3 DISCUSSION

Here we report a method to analyse cell‐free DNA and RNA stained with Pyronin Y by conventional flow cytometry. We applied the method to analyse the cell‐free nucleic acids content of plasma samples of GBM patients. We hypothesized that the measurement of the total nucleic acid content could be a biomarker for GBM, since a previous study described a correlation between the concentration of cell‐free DNA and GBM treatment response (Nørøxe et al., 2019). Indeed, our study shows that the cell‐free nucleic acid content is significantly higher in GBM plasma samples compared to healthy controls. We also analysed the total number of detected events in the plasma samples and found them to be slightly increased in GBM patients. This observation is in line with previous studies that analysed the number and size of EVs isolated from the plasma of GBM patients. They found that the number of EVs is increased in the plasma of GBM patients compared to the plasma of healthy controls (André‐Grégoire et al., 2018; Osti et al., 2019). Furthermore, we found a significant difference in the size of events (violet SSC‐A), with plasma samples of GBM patients showing larger events. Therefore, we calculated a composite *z*‐score of the nucleic acid staining and the size of the events and found that most of the GBM patients have a higher *z*‐score than healthy controls. Taken together, we show that the Pyronin Y staining is a suitable method for detecting differences in the plasma samples of patients.

The described technique could be considered as a potential tool to detect a potential biomarker for the diagnosis and prognosis stratification of glioma by assessing the specificity of the analysis and increasing the diversity of samples used. However, this work presents some limitations. Further control experiments would be necessary to show the specificity of the staining for potential biomarker detection. For example, samples of plasma of patients with different tumour pathologies as well as of patients with inflammatory conditions could be tested. Furthermore, to establish the method as a valid tool for GBM diagnosis, it needs to be tested in a larger cohort. However, we were able to achieve the main goal of this study, which is not to find a biomarker in liquid biopsy of GBM, but to demonstrate that the flow cytometry method based on staining with Pyronin Y works for the detection of cell‐free nucleic acids in both EVs and plasma.

To characterize if the Pyronin Y staining is specific to DNA and RNA under our experimental conditions, we analysed EVs isolated from cell‐conditioned media and plasma. Additionally, we stained free DNA and RNA. A part of the stained EV‐associated DNA and RNA was sensitive to DNase I or RNase A digest. This indicates that some of the nucleic acids are present on the surface of the EVs. This is in line with observations in previous studies that used various methods for DNA detection. Super‐resolution imaging of single EVs isolated from glioma primary cell culture revealed three‐fold more DNA outside than inside EVs (Maire et al., 2021). Analysis of the EV‐associated DNA amount by nano flow cytometry showed a similar result (Liu et al., 2022). Furthermore, analysis of the DNA amount of plasma EVs by qPCR showed that a large part of the EV‐associated DNA was DNase I sensitive (Neuberger et al., 2021). While we observed a specific staining of genomic DNA, the staining of different types of RNA did not show PE+ events. However, we showed that RNA associated with EVs can be stained with Pyronin Y. Therefore, we speculate that the amount, size, and structure of the RNA as well as its association with proteins, particles, or EVs might be different between the samples. This might lead to varying ability to detect the signal by flow cytometry.

A strength of our new method is that it is not only suitable for the analysis of EVs but also for plasma samples. Thus, no information gets lost due to the isolation process of EVs. Furthermore, the direct staining of plasma samples minimizes sample processing and is suitable for very small sample volumes as it requires only 1/100 of sample volume per analysis compared to prior EV isolation. Furthermore, the staining of plasma is highly reproducible with a coefficient of variation of only 6%. Another advantage of the method is that it is accessible to other laboratories as it does not require any specialized equipment but just a conventional flow cytometer. However, the use of conventional flow cytometry also represents a limitation of the study as the size of EVs is at the detection limit of the equipment. The application of a UV laser improves the detection of small vesicles by measuring the violet SSC‐A. But we are still not able to distinguish between cell debris and nucleic acid‐negative EVs because they are all present in the background noise. Therefore, some valuable information about the nucleic acid content of the plasma samples might be missed. The use of a nano flow cytometer would overcome the problem as it has been shown for the DNA analysis of EVs isolated from cell‐conditioned media (Liu et al., 2022). A further limitation of the use of flow cytometers for the analysis of EVs is the detection of ‘swarm effects’ (Welsh et al., 2020; Welsh et al., 2023). We found that the fluorescent signal of staining of genomic DNA and EVs showed a high correlation with nucleic acid concentration and particle count, respectively. Thus, the detection of the signal is in the linear range of the flow cytometer, and we can exclude that background particles are affecting the analysis. Furthermore, we showed that Triton X‐100 treatment reduces the number of events, which confirms the presence of EVs. However, some of the events might be large individual complexes of nucleic acids and proteins that co‐eluted with the EVs in the SEC (Arroyo et al., 2011; Jeppesen et al., 2019; Wei et al., 2017). Furthermore, the EV samples analysed in our study might also contain some free nucleic acids. It has been shown that SEC is not able to efficiently separate cell‐free DNA from the EV fractions. In contrast, density gradient ultracentrifugation can separate the two populations but leads to the loss of EV surface‐associated DNA due to the harsh isolation method (Liu et al., 2022).

Flow cytometry has previously been used to analyse the nucleic acid content of EVs derived from cell‐conditioned media. SYTO™ 16 staining has been used to study EV‐associated DNA (Liu et al., 2022) and SYTO™ RNASelect™ and Quant‐iT™ RiboGreen™ have been used for the analysis of EV‐associated RNA (Fortunato et al., 2021). In contrast, using Pyronin Y, we can simultaneously analyse the DNA and RNA content of EVs. In this study, we apply a flow cytometry‐based method to the analysis of plasma samples. Currently, cell‐free nucleic acids in plasma are analysed by sequencing or PCR‐based methods (García‐Pardo et al., 2022). However, the drawback of these methods is that they are work‐intensive or expensive. A different approach for the analysis of cell‐free DNA is the direct fluorescence assay. It allows to measure the DNA without prior isolation using a fluorometer (Goldshtein et al., 2009). This assay is similarly cost‐effective and simple in application as our proposed flow‐cytometry‐based method. However, our method shows lower inter‐ and intra‐day coefficients of variation. Furthermore, the use of a flow cytometer is advantageous because it not only measures the fluorescence intensity but also the size of events. This gives an additional variable to discriminate between samples. Finally, the use of Pyronin Y allows the detection of the total nucleic acid content and not only of cell‐free DNA.

In summary, this study shows that Pyronin Y staining is a suitable method for the detection of cell‐free nucleic acids in EVs and plasma. If the method is validated and further optimized in future studies, it will be a valuable addition to the existing methods for the analysis of cell‐free nucleic acids in liquid biopsy. An improvement in the analysis of cell‐free nucleic acids could enhance their potential application as biomarkers for cancer.

## 4 METHODS

### 4.1 Patient population

GBM patients (*n* = 15) and healthy controls (*n* = 15) have been enroled at Fondazione IRCCS Istituto Neurologico ‘Carlo Besta’—Milan, Italy––after informed consent. IRB approval has been achieved.

Inclusion criteria: GBM patients: First diagnosis of GBM (WHO grade 4 glioma), age 18–65, unifocal lesions, good functional status (KPS > 70) and candidate for tumour resection followed by standard Stupp protocol. Healthy controls: healthy individuals (not affected by any known pathology that can affect the concentration and protein cargo of plasma‐EVs), matched for age and sex to the classes of patients under study. Exclusion criteria: GBM patients: Candidates for experimental treatments or clinical trials, patients unsuitable for surgery, pregnancy, haematological diseases, other neoplastic diseases (including in anamnesis), other CNS (Central nervous system) diseases (including in anamnesis), contraindications to undergo to magnetic resonance imaging or blood samplings. Healthy controls: Subsequent detection of pathologies, which may affect the concentration and protein cargo of plasma‐EVs, in place at the time of blood collection for the study, pregnancy and contraindication to undergo blood sampling.

### 4.2 Ethics approval and consent to participate

IRB approval has been achieved at Fondazione IRCCS Istituto Neurologico ‘Carlo Besta’ and European Institute of Oncology IRCCS. This study also complied with the guidelines set forth by the Declaration of Helsinki (2008). All patients provided written informed consent for their participation in the study and their identities have been anonymized.

### 4.3 Plasma separation

For establishing the method, single donor human plasma was obtained from Innovative Research (#IPLASNAE10ML), which is plasma that was collected using Na EDTA as an anticoagulant. For the application of the method to analyse the nucleic acid profile of the study population (GBM patients (*n* = 15) and healthy controls (*n* = 15)), whole blood was drawn into ETDA tubes and placed on the benchtop for 15 min. After 15 min, each tube was centrifuged at 2000 × *g* for 10 min at room temperature (RT) to pellet red blood cells. The upper plasma fraction was then collected, transferred to a new sterile tube, and centrifuged again at RT for 10 min at 2000 × *g*. The obtained platelet poor plasma was aliquoted, stored at −80°C and shipped to the European Institute of Oncology IRCCS—Milan, Italy.

### 4.4 Cell culture

U87‐MG cells (ATCC HTB‐14; RRID:CVCL_0022) were maintained and expanded in DMEM (Gibco #11995065) supplemented with 10% (v/v) foetal bovine serum (FBS; Gibco #26140079) and 1x Penicillin‐Streptomycin‐Amphotericin B (Lonza #17‐745E), at 37°C with 5% of CO_2_. For EV production, U87‐MG cells were seeded in 150 cm (Kustanovich et al., 2019) Sarstedt tissue culture dishes in 15‐mL maintenance media at 4 million cells per plate and let attach for 16 h. EV‐depleted media was prepared by 16 h ultracentrifugation at 100,000 × *g* in a Optima™ L‐90K Ultracentrifuge using a Type 45 Ti rotor (k‐factor 133) of DMEM (Gibco #2106302) supplemented with 1% (v/v) FBS and 1x Penicillin‐Streptomycin (Lonza #17‐602F). The cells were washed with 5‐mL EV‐depleted media and then incubated with 15‐mL EV‐depleted media. Cell‐conditioned media was collected after 24 h of incubation and cell count and viability were determined using a trypan blue assay (Invitrogen #T10282). At the time point of media collection, plates contained on average 6*10^6^ cells with a viability of 96%.

### 4.5 EV isolation

Thirty millilitres of U‐87 MG cell‐conditioned media was collected and spun at 1500 × *g* for 10 min to remove cell debris. The supernatant was concentrated to a final volume of about 300 μL using ultrafiltration columns with a 100‐kDa molecular weight cut‐off (Millipore #UFC901024). For EV isolation, 200‐μL concentrated media or 200‐μL human plasma was fractionated using in‐house SEC as described previously (Prieto‐Fernández et al., 2019). Ten fractions of 200 μL and two final fractions of 1.0 mL were collected and stored until analysis at −80°C. Repeated freeze–thaw cycles were avoided.

### 4.6 EV characterization

The size distribution of EV preparations was analysed by nanoparticle‐tracking analysis. A NanoSight LM10 system (Malvern, U.K.) with the fast video capture and particle‐tracking software (NanoSight 3.4) was used. For each preparation, three videos of 60 s each were taken at camera level 9. NTA post‐acquisition settings were kept constant for all samples with a detection threshold of 5. Additionally, the protein concentration of the EV preparations was determined using a Bradford protein assay (Bio‐Rad #5000006).

### 4.7 Immunoblotting

All proteins were analysed under non‐reducing conditions using the NuPAGE system for separation and transfer (Invitrogen). Twenty microlitres of each SEC fraction was mixed with sample buffer and was incubated for 5 min at 37°C, 10 min at 65°C, 15 min at 95°C, and centrifuged at 20,000 × *g* for 1 min. The supernatant was separated on NuPAGE 4%−12% pre‐casted gels (Invitrogen #NP0336BOX) and proteins were transferred to a PVDF membrane (Millipore #IPVH85R). The membrane was incubated overnight at 4°C with the primary antibody (1:1000 in TPBS with 5% BSA), followed by application of the corresponding secondary HRP‐conjugated antibody (1:6000 in TPBS with 5% milk powder) for 45 min at room temperature. Chemiluminescent bands were detected with Clarity Max Western ECL Substrate (Bio‐Rad #1705062) on an ImageQuant LAS 4000 imager (GE Healthcare). Antibodies were purchased from the following vendors: mouse monoclonal antibody against CD63 (H5C6; #NBP2‐42225; RRID:AB_2884028) from Novus, against Flotillin‐1 (#610821; RRID:AB_398140) and CD81 (JS81; #555675; RRID:AB_396028) from BD Biosciences and against CD9 (#MAP1180) from R&D Systems; rabbit monoclonal antibody against COX IV (3E11; #48850) from Cell Signalling. The anti‐mouse (#ab205724) and anti‐rabbit (#ab205718) secondary antibodies were obtained from Abcam.

### 4.8 Cryo‐electron microscopy

EV preparations were directly absorbed onto glow‐discharged holey carbon grids (Quantifoil). Grids were blotted at 95% humidity and rapidly plunged into liquid ethane using the Vitrobot system (Maastricht Instruments). Vitrified samples were imaged at liquid nitrogen temperature using a JEM‐2200FS/CR transmission cryo‐electron microscope (JEOL) equipped with a field emission gun and operated at an acceleration voltage of 200 kV.

### 4.9 Flow cytometry analysis of Pyronin Y staining

A stock solution of 10‐ mg/mL Pyronin Y (Merck #213519) in water was prepared and stored at 4°C protected from light. The working solution was freshly prepared for each experiment. For all steps, DPBS (Gibco #14190250 or #14040117) that was passed through a 0.1‐μm pore filter was used. The 24‐μg/mL Pyronin Y working solution was prepared in a polycarbonate thick‐wall tube (Beckman Counter #362305) and was centrifuged to remove particles for 1.5 h at 220,000 × *g* and at 4°C in an Optima TLX TABLE centrifuge using a TLA‐110 rotor. The supernatant was collected and kept protected from light at 4°C until use. Five microlitres of each SEC fraction or EV preparations containing 1*10^8^ particles was used for each reaction in a final volume of 50‐μL DPBS without Mg^2+^ Ca^2+^. Plasma samples were spun at 2500 × *g* for 10 min at 4°C and the supernatant was diluted 1:100 in DPBS. All reactions were diluted 1:2 with Pyronin Y working solution to a final Pyronin Y concentration of 4 μg/mL for EV and DNA staining and 12 μg/mL for RNA and plasma staining. The reaction was incubated protected from light at room temperature for 20 min. Samples were analysed using a CytoFlex flow cytometer (Beckman Coulter) and the CytExpert software. The fluorescent signal was acquired using a 488‐nm laser and a 585/42 ‐nm band pass filter (PE channel) and a 405‐nm laser and a 450/45‐nm band pass filter (PB channel). Laser powers were adjusted so that the fluorescence intensity was inside the detection range. DNA, RNA and EV samples were measured for 30 s and plasma samples for 60 s. were measured in five technical replicates All samples were analysed at a flow rate of 10 μL/min and event rate setting high activated. Between every sample, the tubing was washed with filtered PBS to avoid carryover of fluorescently positive events. Megamix‐Plus beads (BioCytex #7802/3) were used as a size standard.

### 4.10 Control experiments for Pyronin Y staining

To test the nucleic acid specificity of the Pyronin Y staining, the samples were diluted as described above in a final volume of 50‐μL DPBS Mg^2+^ Ca^2+^. The DNA or RNA of U‐87 MG cells (isolated using the AllPrep DNA/RNA Mini Kit (QIAGEN #80204)) as well as the RNA ladders (Thermo Scientific #SM1831 and NEB #N0363S) were also diluted in DPBS to 50–400 ng of DNA or 1 μg of RNA. The preparations were kept untreated or treated with 0.1‐U/μL DNase I and 1‐mM RNase OUT (Invitrogen #18047019, #10777019) for 15 min at room temperature, with 0.1‐mg/mL RNase A (Thermo Scientific #EN0531) for 30 min at 37°C or with 0.1% (v/v) Triton X‐100 (Merck #T9284‐100ML) for 20 min at room temperature. For the double digest, the samples were first treated with 0.1‐U/μL DNase I for 15 min at room temperature and then with 0.1‐mg/mL RNase A for 30 min at 37°C.

To test the colocalization of EV markers with the Pyronin Y staining, EVs were diluted to EV preparations containing 1*10^8^ particles were used. CD63 antibody or respective IgGs were added to a final dilution of 1:4. The reaction was set up in a total volume of 50‐μL PEB buffer (50‐mM EDTA, 5% BSA in PBS). The reaction was incubated at 4°C for 1 h. Antibodies were purchased from the following vendors: human monoclonal antibody against CD63 conjugated with eFluor450 (H5C6; #48‐0639‐41; RRID:AB_2574022) and mouse monoclonal antibody against IgG1 kappa Isotype Control conjugated with eFluor450 (P3.6.2.8.1; #48‐4714‐82; RRID:AB_1271992) from eBioscience.

For both control experiments, Pyronin Y working solution was directly added to the reactions and the samples were processed as described above.

### 4.11 Statistics

Statistical analysis was performed using the software R (4.1.0) (R Core Team, 2022). Outliers in the technical replicates of plasma samples were removed using box plots. Normal distribution of the data was tested by a Shapiro–Wilk test and equal variance was tested using an F‐test. To analyse the fold change, a one‐sample *t*‐test was applied. A two‐sample *t*‐test was applied for data with normal distribution and equal variance to compare two independent groups. In the case of not normally distributed data, a Mann–Whitney–Wilcoxon *U* test was used. The *z*‐score of the plasma samples for each variable was calculated using the measurements of the control samples as a reference mean and standard deviation. The composite *z*‐score was calculated as the average of the *z*‐scores of the variables.

## AUTHOR CONTRIBUTIONS

J.M.F.‐P. and E.R. designed the study. E.R. performed the experiments and data analysis. E.Z., S.F., M.D.B., F.D.M. and G.P. provided clinal samples. J.M.F.‐P. and E.R. wrote the manuscript. E.Z., G.P., M.D.B., P.H.L. and F.D.M. edited and revised the manuscript. All authors read and approved the final manuscript.

### CONFLICT OF INTEREST STATEMENT

The authors declare no conflicts of interest.

## Supporting information

Supporting Information is available from the Wiley Online Library or from the author.

## Data Availability

The datasets used and/or analysed during the current study are available from the corresponding author on reasonable request.
