# Supplementary material for: Increased levels of circulating cell‐free double‐stranded nucleic acids in the plasma of glioblastoma patients
Source: J Extracell Biol. 2024 Aug 2;3(8):e168. doi: 10.1002/jex2.168 (PMC11294885; doi:10.1002/jex2.168)
Supplement: Supplementary file 1 — Supporting Information is available from the Wiley Online Library or from the author. [file JEX2-3-e168-s001.docx]

*Supplementary figures*

**Increased levels of circulating cell-free double-stranded nucleic acids in plasma of glioblastoma patients**

*Elisabeth Rackles^a^, Elena Zaccheroni^b^, Patricia Hernandez Lopez^a^, Stefania Faletti^b^, Massimiliano Del Bene^c^, Francesco DiMeco^c, d, e^, Giuliana Pelicci^b,f^, Juan M Falcon-Perez^a, g, h,^*

^a^ Center for Cooperative Research in Biosciences (CIC bioGUNE), Basque Research and Technology Alliance (BRTA), Derio, Spain. [erackles.visitor@cicbiogune.es](mailto:erackles.visitor@cicbiogune.es), [phernandez@cicbiogune.es](mailto:phernandez@cicbiogune.es), jfalcon@cicbiogune.es.

^b^ Department of Experimental Oncology, European Institute of Oncology (IEO), IRCCS, 20139, Milan, Italy. [elena.zaccheroni@ieo.it](mailto:elena.zaccheroni@ieo.it), [stefania.faletti@ieo.it](mailto:stefania.faletti@ieo.it), giuliana.pelicci@ieo.it.

^c^ Department of Neurosurgery, Fondazione IRCCS Istituto Neurologico Carlo Besta, Milan, Italy. [massimiliano.delbene@istituto-besta.it](mailto:massimiliano.delbene@istituto-besta.it), fdimecco@istituto-besta.it

^d^Department of Pathophysiology and Transplantation, University of Milan, Milan, Italy.

^e^Department of Neurological Surgery, Johns Hopkins Medical School, Baltimore, Maryland, USA.

^f^ Department of Translational Medicine, University of Piemonte Orientale, Novara 28100, Italy

^g^ Centro de Investigación Biomédica en Red de Enfermedades Hepáticas y Digestivas (Ciberehd), Madrid, Spain.

^h^ Ikerbasque, Basque Foundation for Science, Bilbao, Spain.


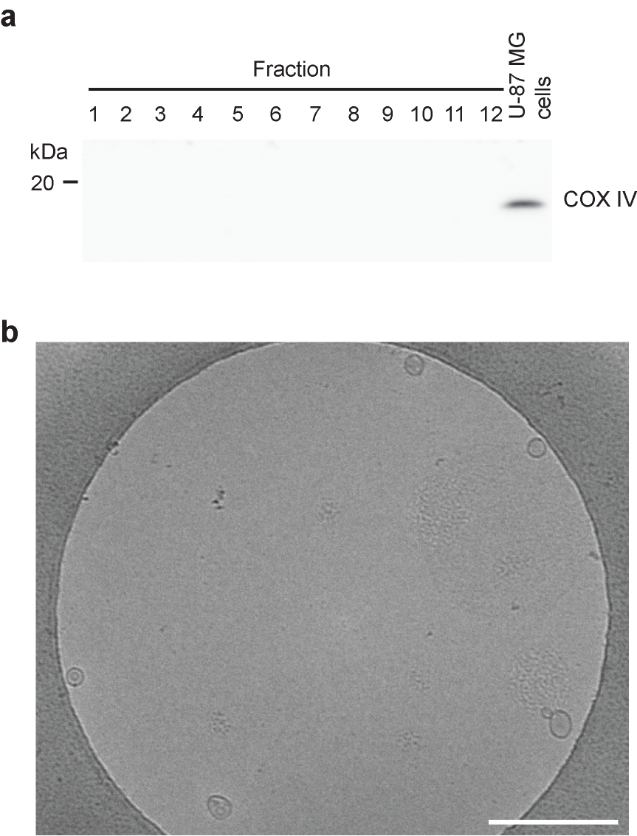


**Supplementary Figure 1**: Characterization of EVs isolated from U-87 MG cell conditioned media by SEC. (A) Immunoblot of the analysis of all SEC fractions. A representative image of three independent isolations is shown (n=3). (B) Representative image of cryo-electron microscopy of EVs present in fraction four. The scale bar represents 500 nm.


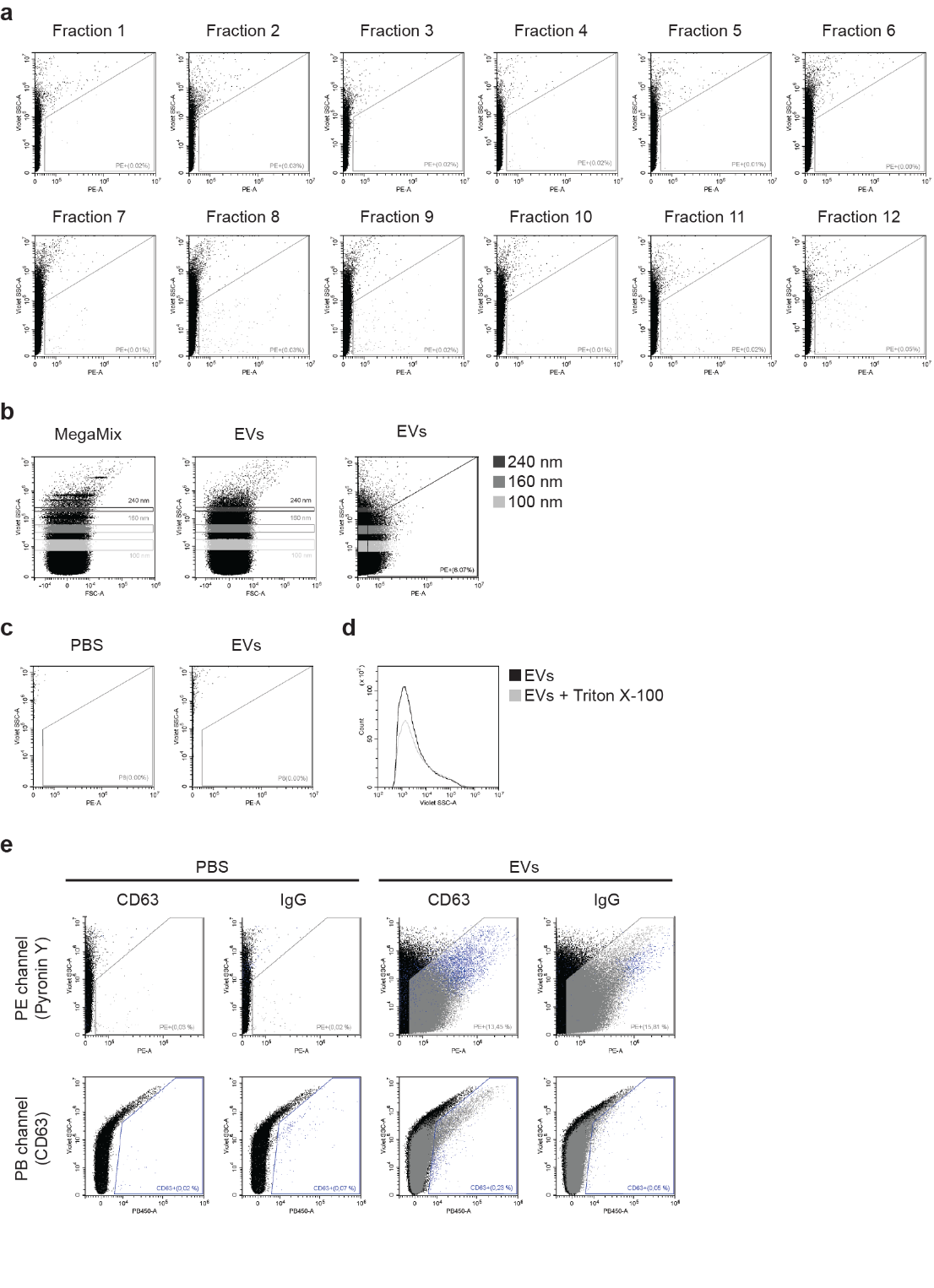


**Supplementary Figure 2**: Control experiments for the analysis of EVs stained with Pyronin Y by flow cytometry. (A) Representative dot plots of all fractions stained with Pyronin Y of a SEC of EV-depleted media (no cells control). (B) Representative dot plot of Megamix-Plus beads. Gates were drawn to compare the violet SSC-A of the beads with the distribution of events of U-87 MG EVs stained with Pyronin Y. (C) Representative dot plots of unstained PBS or U-87 MG EVs. (D) Representative histogram of events of unstained U-87 MG EVs with or without Triton X-100 treatment. (E) Double staining of EVs with Pyronin Y and CD63 antibody. Representative dot plots are shown.


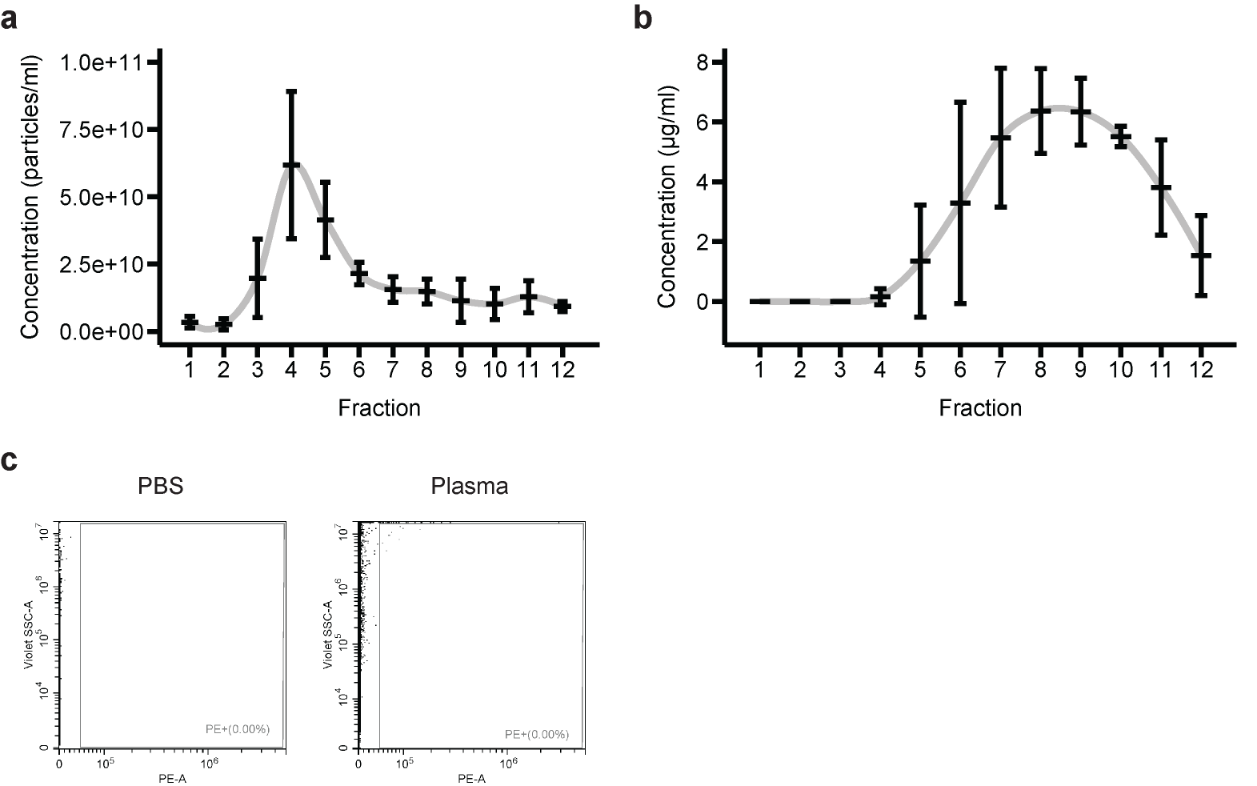


**Supplementary Figure 3**: Characterization of plasma EVs isolated by SEC. (A) Particle concentration of each fraction was measured by NTA. Mean and standard deviation of three plasma samples are shown (n=3). (B) Protein concentration of each fraction measured by Bradford assay. Mean and standard deviation of three plasma samples are shown (n=3). (C) Representative dot plots of unstained PBS or plasma.


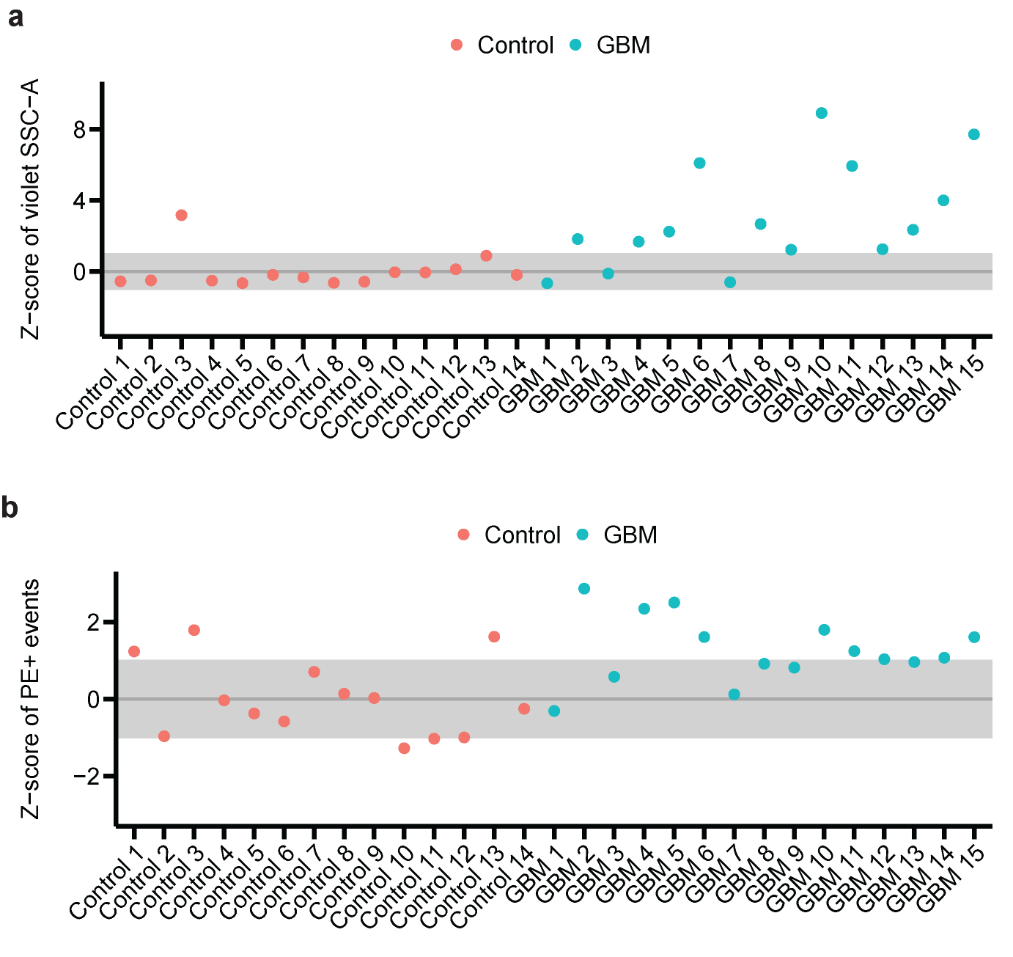


**Supplementary Figure 4**: Pyronin Y staining of plasma samples of GBM patients and healthy controls. (A-B) Plasma samples were stained with Pyronin Y and analysed by flow cytometry. (A) Z-score of the violet SSC-A. (B) Z-score of the number of PE+ events.
